# Unearthing the determinants of digital innovation adoption in the agricultural sector: The role of food security awareness and agricultural experience

**DOI:** 10.1016/j.heliyon.2025.e41695

**Published:** 2025-01-03

**Authors:** David Aboagye-Darko, Peter Mkhize

**Affiliations:** a Department of Information Technology Studies, University of Professional Studies, Accra, Ghana; b Department of Information Systems, College of Science, Engineering, and Technology, University of South Africa, Johannesburg, South Africa

**Keywords:** Digital innovation, Agricultural sector, Food security awareness, Agricultural experience, Ghana

## Abstract

Extant research has widely acknowledged the role of digital innovation as a facilitator of digital transformation, presenting solutions for various challenges in various industries. However, prior research demonstrates inadequate discussions on the determinants of digital innovation adoption for digital transformation in developing countries, particularly in the agricultural sector. To address this gap, this study investigates the effect of food security awareness, innovation characteristics, and the moderating role of agricultural experience on behavioral intention to adopt digital innovation in the agricultural sector. A dyadic model based on diffusion innovation theory and the technology acceptance model is proposed to investigate the phenomenon. This study employed a cross-sectional quantitative approach to investigate the phenomenon based on survey data collected from 207 study participants in Ghana's agricultural sector and the partial least square structural equation modeling technique. The study's findings revealed that personal innovativeness significantly affects food security awareness (β = 0.574; p < 0.000), relative advantage (β = 0.699; p < 0.000), compatibility (β = 0.687; p < 0.000), and complexity (β = 0.312; p < 0.000). In addition, food security awareness (β = 0.336; p < 0.000), compatibility (β = 0.257; p < 0.000), and agricultural experience (β = 0.238; p < 0.003) significantly affect behavioral intention to adopt digital innovation. Furthermore, the study revealed that agricultural experience (β = −0.145; p < 0.036) moderates the relationship between compatibility and behavioral intention. Together, these variables explain 78.9 % of the variance in behavioral intention to adopt digital innovation in the agricultural sector in Ghana. The study contributes to the literature on digital innovation adoption in the agricultural sector in developing countries and proffers actionable insights for practitioners.

## 1 Introduction

According to Ref. [1], one of the major catalysts for digital transformation is the adoption of digital innovation, presenting solutions for challenges in various industries. Consequently, digital innovation has been actively pursued by governments and citizens across various sectors due to its widely acknowledged potential to develop innovative products and services [2]. In addition, digital innovation creates innovative forums involving dynamic sets of actors with different capabilities, inspires new genres of innovation processes, and transforms industries [3]. Due to the potential solutions digital innovation proffers, studies on digital innovation adoption in the agricultural sector in developed countries have focused on leveraging its transformative power [4], not only to improve efficiency and productivity [5] but also to create digital marketplaces that connect stakeholders directly to buyers, partners, and financial institutions [6] to minimize the reliance on middlemen [7]. Unlike developed countries that have successfully adopted digital innovation in their agricultural sectors due to access to advanced technological infrastructure, technical know-how, privacy, and security, their developing counterparts are often underscored by unique challenges such as inadequate access to advanced technologies, and lack of technical know-how [8]. In addition, developing countries usually grapple with limited internet connectivity in rural areas and small-scale farming locations [9]. Furthermore, while research developed countries highlight the use of drones and satellite imagery to enable farmers to evaluate the health of crops, detect pests and diseases, and improve pesticide and fertilizer usage [10], developing countries lag in terms of the adoption of these innovations. These contrasting findings emphasize the need for context-specific studies, especially in developing countries, where the facilitating environment for digital innovation adoption differs remarkably. Therefore, it is essential to investigate the determinants of digital innovation adoption for digital transformation in the agricultural sector in developing countries. The significance of this matter is crucial because although existing evidence supports the idea that adopting digital innovation engenders new pathways for value creation [11], the varying levels of success, in terms of adoption, across developing and developed countries make the determinants of digital innovation adoption in developing country contexts unclear.

Existing studies on digital innovation adoption in the agricultural sector in developing countries have focused on demographic factors, adopter categories, remittances, and socio-economic factors such as farm size and location [[12], [13], [14]]. However, the effect of food security awareness and the moderating role of agricultural experience on the intention to adopt digital innovation has been under-explored. For instance, while exploring the adoption of digital innovation in the agricultural sector [12], focused on demographic characteristics and agri-cooperatives, however, the effects of food security awareness and the moderating role of agricultural experience were not examined. Similarly [13], studied digital innovation adoption in the agricultural sector, focusing on socioeconomic antecedents and adopter categories, without attention to how food security awareness and agricultural experience influence intention to adopt digital innovation. Furthermore [14], studied the effects of demographic, technological, and socio-economic determinants of digital innovation adoption. However, their study did not examine food security awareness and the moderating effect of agricultural experience on digital innovation adoption.

Food security is a serious challenge and, hence, is gaining attention in academia and industry [15]. Similarly [16], suggests that food security is captured in many current policy agendas compared to past policy agendas. This has led to the global call for food security awareness to strengthen rural-urban linkages and facilitate the achievement of food security [15]. Furthermore, an individual's agricultural experience influences his or her assessment of the challenges and opportunities within the sector. In the context of digital innovation adoption, these issues raise intricate questions about the effect of food security awareness and the moderating role of agricultural experience on user behavior in the agricultural sector. By investigating this phenomenon, this study seeks to advance knowledge on the implications of food security awareness and agricultural experience on digital innovation adoption in the agricultural sector in developing countries. In addition, this study complements existing literature on the determinants of digital innovation adoption for digital transformation from a dyadic theoretical perspective. Consequently, this study builds on the Diffusion of Innovations (DOI) theory and the Technology Acceptance Model (TAM). The DOI theory assesses the characteristics of digital innovations, while TAM examines the cognitive dimensions concerning the intention to adopt digital innovation. Thus, the overarching research question is: *what are the factors that influence digital innovation adoption in the agricultural sector in developing countries?*

To address this research question, this study investigates the determinants of digital innovation for digital transformation in Ghana's agricultural sector based on the DOI and TAM theories. Data gathered from study participants in Ghana were analyzed using partial least squares structural equation modeling (PLS-SEM). Given that the authors sought to examine complex relationships that reveal the factors that influence the adoption of digital innovation in Ghana's agricultural sector, PLS-SEM was deemed appropriate because of its robustness. Secondly, PLS-SEM was adopted because it can handle skewed sample distribution and small sample size compared to other analytical techniques such as LISREL, AMOS, and EQS [17,18].

The research is organized as follows. The next section discusses the literature, the hypotheses, and the research model. The fourth section presents the methodology. This is followed by results and analysis in the fifth section. The sixth section presents a discussion, theoretical contributions, and practical implications. Section seven concludes the study and discusses study limitations.

## 2 Literature review

The pervasive digitalization and technological advancement allow various industries to apply digital technology, fully or partly, to individual or organizational activities which leads to the development of innovative processes [19]. The application of digital technologies to create business processes or activities that lead to changes in business models and market offerings is known as digital innovation [3]. Digital innovation enables organizations and industries to create and exploit value-adding activities that are digitally enabled via the use of digital technologies to provide flexibility and fluidity of processes and outcomes [1]. Having realized the potential of digital innovation to transform industries and organizations, allowing for the creation of an interconnected digital environment [3], the agricultural sector in both developed and developing countries is making significant investments in the adoption of digital innovations. The adoption of digital innovation refers to the decision to use digital innovation in a particular context. The idea behind adopting digital innovation in the agricultural sector originated from the understanding that it is a significant facilitator of entrepreneurial development and new economic opportunities [20].

In the context of digital innovation adoption in the agricultural sector, developed countries have adopted and integrated digital innovation, successfully leveraging experience and global trends such as food security awareness, while their developing counterparts face challenges in this regard [8]. The challenge of inadequate infrastructure including limited access to internet connectivity and reliable power supply, which are essential for digital technologies, is more pronounced in developing countries [8]. In addition, the initial cost of obtaining a digitized agricultural environment due to digital innovation adoption may be high for developing countries, which have inadequate financial resources [21].

Food security awareness and agricultural experience are essential factors in understanding the digital innovation adoption lacuna between developing and developed countries. With a high level of food security awareness, individuals are more likely to perceive the potential opportunities and benefits of adopting digital innovation, such as streamlined processes while ensuring food security [15]. Similarly, individuals with extensive agricultural experience may have insights into how digital innovations can resolve context-specific challenges or improve existing agricultural practices [22]. However, strong claims cannot be made about the effect of food security awareness and agricultural experience on digital innovation adoption in the agricultural sector in developing countries. Most studies examine demographic, technological, and socio-economic antecedents to invalidate or validate the determinants of digital innovation adoption (e.g., Refs. [[12], [13], [14]]), overlooking agricultural factors that could shape adoption. This research extends the literature on digital innovation adoption in the agricultural sector in developing countries by examining the effect of food security awareness on the intention to adopt digital innovation and the moderating effect of agricultural experience on the relationship between innovation characteristics and behavioral intention. The study builds on the Diffusion of Innovations (DOI) theory and the Technology Acceptance Model (TAM) and proposes a dyadic model to investigate the factors that influence digital innovation adoption in the agricultural sector in developing countries.

Previous research (e.g., Refs. [14,21,23]) has primarily explored the determinants of digital innovation adoption in the agricultural sector in developing countries without investigating the impact of food security awareness and agricultural experience. For instance Ref. [23], analyzed the factors that shape digital innovation for rice cultivation among farmers in Ghana and revealed that socioeconomic factors such as farm size, and access to credit significantly affect smallholder farmers' decision to adopt digital innovations. Similarly [14], explored the determinants of smallholder farmers' adoption of digital innovation in Ethiopia. Their findings revealed that sex, age, education, farm size, and access to credit significantly affect smallholder farmers' decision to adopt digital innovations. Also [21], explored the factors that influence farmers' decisions to adopt post-harvest innovations in Tanzania. The authors revealed that off-farm incomes, bank account/mobile money ownership, and group membership are key determinants of digital innovation adoption in the agricultural sector. However, prior research ([14,21,23]) has not shed light on the multifaceted ways in which food security awareness and agricultural experience facilitate or impede digital innovation adoption in the agricultural sector in developing countries. Given that food security awareness enhances users' inclination to adopt digital channels to improve household food security status [15], while agricultural experience informs users’ assessment of the challenges and opportunities of adopting technological platforms [22], it is important to address this gap in the literature. Hence, there is a need to understand the effect of food security awareness and agricultural experience on digital innovation adoption, a lacuna this research seeks to fill. Table 1 presents sample prior studies on digital innovation adoption in the agricultural sector in developing countries.

**Table 1:** Sample prior studies.

Studies	Research objective	Theoretical underpinnings	Study context	Constructs
Ayisi et al. (2022)	To examine the effect of socio-economic attributes on smallholder farmers' innovativeness in the adoption of digital innovations in Ghana. To investigate the innovative adopter categories among smallholder farmers in Ghana	Diffusion of innovations (DOI) theory	Smallholder farmers in Ghana	• Formal education • Gender • Income • Adopter category – innovators, early adopters, early majority, late majority, and laggards
Mutungi et al. (2023)	To explore the factors that influence farmers decisions to adopt post-harvest innovations in Tanzania To examine how digital innovation contributes to food security	–	Farmers in Tanzania	• Farm size • Access to credit • Off-farm incomes • Bank account/mobile money ownership • Group membership
Udimal et al. (2017)	To analyze the factors that shape digital innovation for rice cultivation among farmers in Ghana	–	Farmers in the Northern region in Ghana	• Farm size • Credit access • On-farm demonstration • Tractor ownership • Family labor • Profit orientation
Workie and Tasew (2023)	To explore the determinants of smallholder farmers' adoption of digital innovation for malt barley packages in Ethiopia	Diffusion of innovations (DOI) theory	Smallholder farmers in Ethiopia	• Demographics - sex, age, education, • Socio-economic factors - farm size, family size/labor, income, assets, extension contact, access to credit, market, inputs, information, location, membership, and position in local cooperatives, and • Technological factors - relative advantage, compatibility, complexity, trialability, and divisibility

### 2.1 Diffusion of innovations theory

The Diffusion of Innovation (DOI) theory is essential for understanding how innovations are adopted and spread in societies. The underlying assumption of DOI theory is that the spread of innovation within societies is not random [24]. Instead, Rogers suggests that various factors influence the spread of innovation and follow a unique pattern [25]. The central thesis of DOI theory suggests that individuals or organizations, known as adopters, accept innovation at different stages. In addition, DOI theory categorizes adopters into five groups: innovators, early adopters, early majority, late majority, and laggards [25]. Each adopter plays a unique role in the process of innovation diffusion. While innovators and early adopters demonstrate receptiveness toward new ideas, the majority and laggards are more cautious when embracing new ideas. Also, DOI theory posits that the decision-making process that characterizes the diffusion of innovations includes knowledge, persuasion, decision, implementation, and confirmation [25]. Various factors influence each stage of the decision-making process that characterizes the diffusion of innovations. These factors determine whether or not an individual or an organization is progressing toward the adoption of the innovation.

Moreover, the diffusion process is affected by social systems, the communication channels, and the perceived characteristics or attributes of the innovation. DOI theory suggests that the perceived attributes of innovation, such as observability, compatibility, perceived relative advantage, trialability, and simplicity, are more likely to result in innovation adoption [26]. Furthermore, DOI theory acknowledges the significance of change agents/decision-makers and opinion leaders in expediting the diffusion of innovations. Change agents/decision-makers and opinion leaders bridge communication gaps and accelerate the process of innovation diffusion. Opinion leaders refer to individuals within a societal network who are influential [25]. Opinion leaders are usually among the early adopters of innovation. On the other hand, change agents/decision-makers refer to individuals within a social system who actively facilitate the adoption of innovation [25].

The diffusion of innovations theory suggests that individuals respond differently to adopting innovations. Such disparity in response to innovation is referred to as an individual's innovativeness [27]. [28] advanced a new measurement for personal innovativeness, reflecting an individual's innate propensity towards adopting new information technology. Also, the authors suggest that an individual's cognitive interpretation of information technology is determined by factors related to the individual. Personal innovativeness refers to the degree to which individuals are willing to adopt digital innovation or a new technology [28]. Individuals who demonstrate high levels of personal innovativeness tend to have positive perceptions of digital innovation, particularly regarding compatibility, relative advantage, usefulness, and ease of use.

### 2.2 Technology acceptance model

[29] proposed the Technology Acceptance Model (TAM) which has been widely used to unearth the factors that shape technology adoption in various contexts. Also, TAM was adapted from the Theory of Reasoned Action (TRA) in the information systems discipline. The underlying assumption of TAM is that perceived ease of use and perceived usefulness are key determinants of users’ intention to adopt technology. In addition [30], further argued that perceived ease of use directly affects perceived usefulness. Perceived usefulness is defined as the level to which a user believes a technological system would improve job performance, while perceived ease of use refers to the level to which users believe using a technological system would require less effort [30]. Furthermore, perceived usefulness directly affects behavioral intention, while behavioral intention directly affects actual usage [29]. Hence, the TAM constructs are as follows: perceived usefulness, perceived ease of use, behavioral intention, and actual usage.

### 2.3 Hypotheses development

**Fig. 1:**
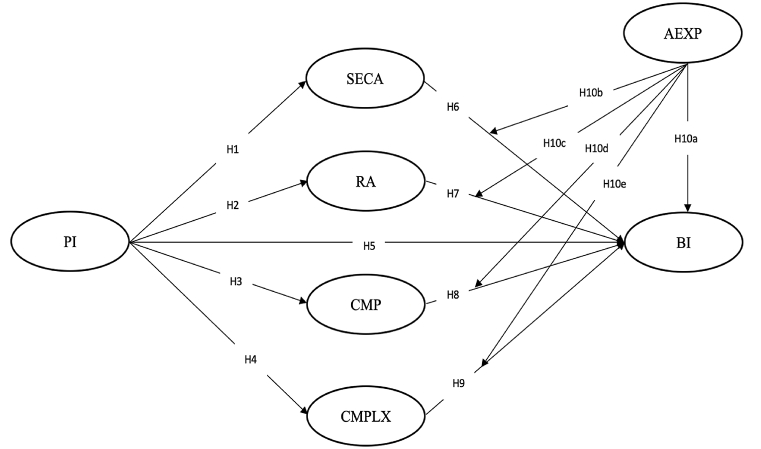
Research model.

Source: Authors' construction

This study builds on the DOI theory and TAM and proposes a dyadic research model to unearth the factors that influence digital innovation adoption in the agricultural sector in developing countries. DOI theory captures the process by which innovation spreads within a society [25], which is particularly relevant in the agricultural sector where compatibility with existing practices is essential. For example, the compatibility of malt barley technology with agricultural practices has enhanced its adoption among smallholder farmers in Ethiopia [14]. On the other hand, TAM emphasizes perceived ease of use and perceived usefulness, which are evident in agricultural settings where the usefulness and ease of use of technology tools must meet the needs of agricultural operations. For example, in the agricultural sector, perceived usefulness drives farmers' intention to adopt green production technologies in agricultural contexts [31]. Therefore, combining these theories provides higher explanatory accuracy than explaining the phenomenon based on either DOI theory or TAM [32,33]. Furthermore [34], suggest that the complexity and relative advantage innovation attributes of the DOI theory correspond to perceived ease of use and perceived usefulness constructs associated with TAM [29]. Following this line of research, this study focuses on relative advantage and complexity, which mirrors the perceived usefulness and ease of use associated with TAM. Therefore, the conceptual model's constructs are personal innovativeness, relative advantage, compatibility, complexity, behavioral intention, food security awareness, and agricultural experience. The conceptual framework of the study is presented in Fig. 1, while the definitions and abbreviations of the conceptual framework's constructs are presented in Table 2.

**Table 2:** Research model's constructs, abbreviations, and definitions.

Construct	Abbreviation	Definition
Personal innovativeness	PI	Personal innovativeness refers to the degree to which individuals are willing to adopt digital innovation (Agarwal & Prasad, 1998)
Food security awareness	SECA	Food security awareness refers to an individual's knowledge and understanding regarding continuous access to safe, nutritionally adequate, and sufficient food (Maxwell, 1996). That is, access to safe, nutritious, and safe food to ensure a healthy and productive life without compromising the safety and quality of food consumed (Anderson & Cook, 1999)
Relative advantage	RA	Relative advantage refers to the perceived benefits of digital innovation compared to existing practices (Rogers, 2003)
Compatibility	CMP	Compatibility refers to the perceived alignment between digital innovation and existing practices, values, and needs (Rogers, 2003)
Complexity	CMPLX	Complexity refers to the perceived difficulty associated with understanding and using digital innovation (Rogers, 2003).
Agricultural experience	AEXP	Agricultural experience refers to an individual's familiarity and years of experience or practice in the agricultural sector (Danso-Abbeam et al., 2020; Anum et al., 2022). Agricultural experience is a proxy for an individual's years of experience or practice in the agricultural sector (Danso-Abbeam et al., 2020).
Behavioral intention	BI	Behavioral intention refers to the likelihood that an individual intends to perform a behavior, such as digital innovation adoption (Davis, 1989).

#### 2.3.1 Personal innovativeness, food security awareness, relative advantage, compatibility, behavioral intention, and complexity

[28] extended the applicability of the concept of “innovativeness” to the information system discipline by conceptualizing Personal Innovativeness (PI) as a variable that accounts for the degree to which individuals are willing to adopt digital innovation. Individuals who demonstrate a higher degree of PI tend to develop positive perceptions about digital innovation regarding relative advantage, complexity, and compatibility. Also, individuals with high levels of innovativeness develop positive intentions toward the adoption of digital innovation [35]. [36] suggest that PI positively affects relative advantage and behavioral intention in technology adoption. Similarly [27], posit that PI has a positive effect on compatibility, while [37] suggests that PI significantly influences an individual's perception of the effort required to use digital innovations (i.e., complexity). Moreover [37,38], and [35] suggest that personal innovativeness directly affects behavioral intention.

Furthermore, individuals with a higher level of PI tend to demonstrate higher adaptability to technological change, intellectual curiosity, and a proactive engagement with digital innovations. This intellectual curiosity goes beyond exploring only the potential of digital innovations to proactively seeking information on how it can be used to ensure food security due to their food security awareness. However, inadequate empirical evidence to affirm this assertion suggests that there is a gap that further studies need to fill.

Extending the research stream of PI within the context of digital innovation adoption in Ghana's agricultural sector, this study defines PI as the willingness of individuals to adopt digital innovation to improve agricultural practices. Individuals with a higher degree of innovativeness are more likely to perceive digital innovation as having significant benefits than existing agricultural practices. This is because individuals with higher innovativeness are open to digital innovation and perceive it as providing benefits over existing practices [36]. Also, individuals with higher innovativeness are more likely to perceive digital innovation as aligning with their existing practices and preferences [27]. Moreover, individuals with higher innovativeness demonstrate higher confidence in their ability to use digital innovation, perceiving the complexity of digital innovation as surmountable rather than an obstacle [37]. Highly innovative individuals' problem-solving and information-seeking nature cause them to seek digital innovations to improve food security and agricultural practices. Also, the specific characteristics of individuals in the agricultural sector, including PI, influence food security awareness [15]. Therefore, individuals with high innovativeness are more likely to adopt digital innovation due to their perception of its benefits and potential to enhance food security. Against this backdrop, the following hypotheses are proposed:

H1 *Personal innovativeness significantly influences food security awareness.*

H2 *Personal innovativeness significantly influences relative advantage.*

H3 *Personal innovativeness significantly affects compatibility*

H4 *Personal innovativeness significantly influences complexity.*

H5 *Personal innovativeness significantly influences behavioral intention*

#### 2.3.2 Food security awareness and behavioral intention

Food security awareness refers to an individual's knowledge and understanding regarding continuous access to safe, nutritionally adequate, and sufficient food [39]. That is, access to safe, nutritious, and safe food to ensure a healthy and productive life without compromising the safety and quality of food consumed [40]. Moreover [15], suggest that an individual's food security awareness enhances their household food security status, particularly in African countries. Hence, food security awareness can encourage positive behavior regarding an individual's willingness to adopt digital innovation to improve food security and agricultural practices. However, inadequate empirical evidence to affirm this assertion suggests that there is a gap that further studies need to fill. In the context of the adoption of digital innovation in Ghana's agricultural sector, individuals with sufficient knowledge and understanding of food security will likely develop intentions to embrace practices that promote food security, including the adoption of digital innovation. This is because digital innovation improves agricultural productivity and facilitates the development of efficient supply chains and sustainable food systems [20]. When an individual's food security awareness increases, he or she develops a more positive intention toward adopting digital innovation due to the significant role digital innovation plays in promoting food security. Hence, it is argued that:

H6 *Food security awareness significantly influences behavioral intention.*

#### 2.3.3 Relative advantage and behavioral intention

Relative advantage refers to an individual's perception of the benefits of digital innovation over existing practices or alternatives [25]. Relative advantage encompasses an individual's perception of the benefits and advantages of adopting digital innovation [41]. [42] argue that relative advantage is an essential predictor of digital innovation diffusion [43,44]. suggest that relative advantage positively affects behavioral intention.

In the context of the adoption of digital innovation in Ghana's agricultural sector, relative advantage reflects individuals' perception of the advantages and benefits of digital innovation over existing agricultural alternatives or practices. When individuals perceive that digital innovation provides significant benefits compared to current practices, they are more likely to develop intentions to adopt it [45]. This is because such individuals are motivated by the benefits and positive outcomes that characterize the adoption of digital innovation. As a result, a higher perception of relative advantage will lead to stronger intentions to adopt digital innovation. Therefore, it is argued that:

H7 *Relative advantage significantly affects behavioral intention.*

#### 2.3.4 Compatibility and behavioral intention

Compatibility refers to the perceived alignment between digital innovation and existing practices, values, and needs of potential adopters [25]. That is, the perceived alignment between digital innovation and existing practices, values, and needs. Compatibility positively affects behavioral intention [46,47]. Compatibility influences how an individual perceives that digital innovation aligns with existing practices within a social system [25].

In the context of digital innovation adoption in Ghana's agricultural sector, compatibility is defined as an individual's perception that digital innovation aligns with existing agricultural practices and values [48]. When individuals perceive that digital innovation aligns with agricultural practices, they are more likely to develop positive intentions toward adopting it [49]. This alignment influences their perception regarding the coherence and ease of integrating digital innovation into their work routines, minimizing perceived hindrances and increasing the likelihood of intention to adopt digital innovations. Therefore, it is hypothesized that:

H8 *Compatibility significantly influences behavioral intention.*

#### 2.3.5 Complexity and behavioral intention

Complexity is the perceived difficulty of understanding and using digital innovation [25]. That is an individual's perception of how challenging it is to use digital innovation [50]. suggest that complexity negatively affects the adoption of digital innovation. Thus, when an individual perceives that adopting digital innovation is a complex endeavor, they are more likely to develop intentions to reject it ([49,51]). As a result, an individual's perception of complexity associated with digital innovation adoption can act as a barrier, minimizing their intention to adopt it. In the context of the adoption of digital innovation in Ghana's agricultural sector, complexity refers to the perceived difficulty of understanding and using digital innovation for agricultural activities. When an individual perceives that the adoption of digital innovation is a complex endeavor, he or she is more likely to develop an intention to reject it. On the other hand, when individuals perceive low complexity associated with digital innovation adoption, they are likely to believe digital innovation is easy to understand and use, increasing their intention to adopt it. Hence, this study posits that:

H9 *Complexity significantly influences behavioral intention.*

#### 2.3.6 Agricultural experience and behavioral intention

Agricultural experience refers to an individual's familiarity and years of experience or practice in the agricultural sector [22,52]. An individual's practical experience influences his or her assessment of the challenges and opportunities regarding digital innovation adoption in the agricultural sector [22]. When an individual has sufficient agricultural experience, he or she is more likely to develop an intention to adopt digital innovation that conforms to his or her understanding of agricultural practices. Also, an individual's understanding and practical insights regarding agricultural activities empower them to recognize the potential benefits of digital innovation [22].

In the context of digital innovation adoption in Ghana's agricultural sector, individuals largely depend on their understanding, familiarity, and practical knowledge of agricultural practices based on their years of experience in the agricultural industry to assess the potential benefits associated with digital innovation adoption. However, the literature on digital innovation adoption in the agricultural sector [[53], [54], [55]] has been silent on the effects of agricultural experience on the connection between digital innovation characteristics and behavioral intention. Without an understanding and practical knowledge of agricultural activities, individuals cannot adequately assess the potential benefits and challenges associated with the adoption of digital innovation in the agricultural sector. Thus, it is essential to understand the effect of agricultural experience on the connection between digital innovation characteristics and behavioral intention.

Based on the aforementioned, the following hypotheses are proposed:

H10a *Agricultural experience significantly influences behavioral intention.*

H10b *Agricultural experience moderates the relationship between food security awareness and behavioral intention.*

H10c *Agricultural experience moderates the relationship between relative advantage and behavioral intention.*

H10d *Agricultural experience moderates the relationship between compatibility awareness and behavioral intention.*

H10e *Agricultural experience moderates the relationship between complexity awareness and behavioral intention.*

## 3 Methodology

### 3.1 Study population

The population of the study is the collection of people, elements, or phenomena that a researcher seeks to make inferences about [56]. A total of 4286 people were employed in the 261 district-level government agricultural agencies (known as the District Department of Agriculture) responsible for improving the impact and reach of agricultural initiatives to effectively address the diverse needs of organizations and individuals in the various districts in Ghana, under the auspices of the Ministry of Food and Agriculture (MoFA) [57]. This study focused on this population for two reasons: 1) The District Department of Agriculture initiate localized efforts in promoting the adoption of digital innovations and sustainable agricultural practices in 261 districts in Ghana. 2) The District Department of Agriculture is mandated to improve the impact and reach of agricultural initiatives to effectively address the diverse needs of organizations and individuals in the various districts in Ghana [57]. By adopting this decentralized approach, MoFA acknowledges the significance of localized efforts in promoting sustainable agricultural practices and the need for uniquely suited responses to the opportunities and challenges in various districts. Therefore, individuals at the District Department of Agriculture can be considered the linchpin in actualizing agricultural initiatives, interventions, and strategies that address the specific demands of various districts in Ghana's agricultural sector.

A purposive sampling technique was adopted to enable the inclusion of study participants who are knowledgeable and have expertise that aligns with the focus of the study. Also, the purposive sampling technique enabled the researcher to focus on study participants with unique experiences and detailed information about Ghana's agricultural sector, where resources and time were constrained [58]. The criteria for selecting the study participants were that, firstly, they must be workers in Ghana's agricultural sector, and secondly, they should have had experience with digital innovations in the agricultural sector.

[59] rule to determine the sample size in a scientific inquiry was adopted. [59] rule suggests that the structural or research model should be examined to identify the highest number of structural paths pointing to a particular construct. After identifying the highest number of structural paths that point to a particular construct, the sample size should be 30 times (30x) that number. That is an acceptable sample size, according to Ref. [59]. Hence, the sample size should be 30 times the highest number of structural paths that point to a particular construct. Behavioral intention (BI) was the construct with the highest number of structural paths (a total of six) pointing to it. That is 6 x 30 = 180. Therefore, going by the 30 times rule proffered by Ref. [59], a sample size of 180 was considered sufficient for this study.

Although the determined sample size was 180, the researcher administered 250 questionnaires through an online survey. Given that questionnaires in scientific inquiries are sometimes characterized by incomplete responses, unexpected contingencies, and non-responses [60], the researcher administered 250 questionnaires to mitigate such risks. Also, administering more questionnaires than the determined sample size improved the final sample's reliability and representativeness [60]. Furthermore, it improved the quality and robustness of the study by reducing the impact of missing data [60].

### 3.2 Data collection method

The data was collected using an online questionnaire comprising 29 items. The study participants were intercepted from October 2023 to January 2024. The data collection instrument was divided into two sections A and B. Section A solicited responses on the demographic profile, and the types of digital innovation used. Section B solicited data on the hypothesized relationships of the study. The questionnaire was developed using a Likert scale with seven possible outcomes (a 7-point Likert scale), ranging from Strongly disagree to Strongly agree [61]. Measurement items in Section B were adapted from empirically validated constructs in extant literature. Items measuring personal innovativeness were adapted from Ref. [28], and items measuring behavioral intention were adapted from Ref. [29]. Also, items measuring complexity, relative advantage, and compatibility were adapted from Ref. [25]. Furthermore, items measuring context-specific variables such as food security awareness and agricultural experience were adapted from Refs. [15,52] respectively.

The study adopted a multi-sequential approach in the research design. To begin, the survey was reviewed by a professor in information systems, two consultants in leading agri-tech companies in Ghana, and a statistician. Their feedback helped refine the data collection instrument. Subsequently, the online questionnaire was piloted. A total of 40 responses were collected to assess the questionnaire's appropriateness. Next, study participants were invited to engage in the survey. To ensure that only eligible respondents participated in the study, a condition was set to end the study for respondents who were not involved in Ghana's agricultural sector. Participation in the survey was voluntary. Also, participants were assured of confidentiality and anonymity. In all, 207 responses obtained were considered acceptable for the analysis. This study drew on Kolmogorov–Smirnov's [62] rule to test for bias between the data collected for the pilot test and the primary data collected for the study. The sample distribution of the two data sets was compared and did not differ statistically, revealing the absence of equality continuous bias. Also, common method bias was assessed by drawing on Harman's one-factor test [63]. The eigenvalues of the study's variables were greater than one, indicating the absence of common method bias.

## 4 Analysis

The descriptive analysis was conducted using SPSS software to examine the demographic characteristics of respondents. This was followed by the analysis of the measurement and structural model using SmartPLS version 4 presented in sections 5, 6 respectively. The measurement and structural model were analyzed using partial least square structural equation modeling (PLS-SEM). Given that the authors sought to examine complex relationships that reveal the factors that influence the adoption of digital innovation in Ghana's agricultural sector, PLS-SEM was deemed appropriate because of its robustness.

### 4.1 Demographic characteristics

The demographic characteristics of the respondents, as presented in Table 3, suggest that most respondents were males (69.1 %) compared to females (30.9 %). These findings imply that this study is not gender biased as it accounts for both genders. Also, a possible reason for the male dominance in the study could be that more male employees responded to the questionnaire than female participants, and not necessarily because more males were employed than females.

**Table 3:** Demographic characteristics of respondents.

Demographic characteristics	Level	Frequency (n)	Percentage (%)
Gender	Male	143	69.1 %
	Female	64	30.9 %
Age	18–30	163	78.7 %
	31–40	40	19.3 %
	41–50	3	1.5 %
	51–60	1	0.5 %
Qualification	Bachelors	128	61.8 %
	Postgraduate	49	23.7 %
	Professional	10	4.8 %
	Other	20	9.7 %

Also, most respondents were between 18 and 30 (78.7 %). This age group was followed by respondents between the ages of 31 and 40 (19.3 %), 41 and 50 (1.5 %), and 51 and 60 (0.5 %). These findings suggest that young adults dominate the study sample. This finding corroborates with [64], which indicates that increased productivity in Ghana's agricultural sector relies heavily on the youth, who constitute approximately 20–30 % of the nation's active population. Furthermore, the findings of the educational qualification distribution of the study sample suggest that holders of bachelor's degrees (61.8 %) dominate the study sample. This was followed by postgraduate degrees (23.7 %), other qualifications (9.7 %), and professional qualifications (4.8 %). These findings imply that most of the respondents of the study sample in the district-level government-regulated agricultural agencies possess basic educational skills and knowledge.

### 4.2 Type of digital innovation

The study's findings in Table 4 suggest that most respondents in the study sample leveraged multiple digital innovations (81.1 %) to achieve integrated digital solutions in Ghana's agricultural sector. In addition, a substantial number of respondents (10.2 %) use mobile applications. This was followed by the use of social media networks (5.3 %), artificial intelligence assistants (1.5 %), and web applications (0.9 %). Furthermore, cloud computing and other digital innovations recorded 0.5 % each.

**Table 4:** Type of digital innovation used.

Type of digital innovation	Frequency (n)	Percentage (%)
Mobile applications	21	10.2 %
Artificial intelligence assistants	3	1.5 %
Cloud computing	1	0.5 %
Web application software	2	0.9 %
Social media networks	11	5.3 %
Other	1	0.5 %
Multiple digital innovations	168	81.1 %

These findings imply that Ghana's agricultural sector is leveraging multiple digital innovations to improve its operations and service delivery, indicating the emergence of a positive approach towards modernization and digital transformation. In addition, the use of mobile applications provides user-friendly and accessible mobile solutions to facilitate access to agricultural extension services, market opportunities, and information. Furthermore, the adoption of artificial intelligence assistants and social media networks indicates a growing reliance on digital innovation for communication, agricultural solutions, and decision-making.

## 5 Measurement model

The validity and reliability of the multiple-item scale of the constructs were assessed using the confirmatory factor analysis. Drawing on the principle of a minimum threshold of 0.70 proffered by Ref. [17], the study findings revealed that the values of the item loading, as presented in Table 5, span from 0.708 to 0.929, except four items (AEXP4, SECA1, SECA2, and BI2) which obtained factor loadings below the required threshold. In addition, these four variables affected the reliability and validity of the study's findings, hence, were removed from the final model. According to Ref. [65], factor loadings below the required threshold can reduce the reliability of the construct, introducing noise and undermining the measurement clarity. Thus, removing factor loadings below the required threshold can provide reliable and valid reflections of the theoretical constructs. In all, the findings demonstrate a good fit concerning the measurement model.

**Table 5:** Construct validity and reliability (Quality criterion).

Constructs	Items	Item Loadings	Cronbach's alpha	Composite reliability (rho_a)	Composite reliability (rho_c)	Average variance extracted (AVE)
AEXP	AEXP1	0.802	0.891	0.896		0.736
	AEXP2	0.882				
	AEXP3	0.887			0.893	
BI	BI1	0.867	0.923	0.924		0.801
	BI3	0.908				
	BI4	0.908			0.923	
CMP	CMP1	0.851	0.914	0.916		0.728
	CMP2	0.870				
	CMP3	0.882				
	CMP4	0.809			0.915	
CMPLX	CMPLX1	0.708	0.839	0.841		0.568
	CMPLX2	0.775				
	CMPLX3	0.753				
	CMPLX4	0.778			0.840	
PI	PI1	0.786	0.885	0.893		0.668
	PI2	0.735				
	PI3	0.875				
	PI4	0.865			0.889	
RA	RA1	0.867	0.926	0.927		0.759
	RA2	0.881				
	RA3	0.890				
	RA4	0.848			0.927	
SECA	SECA4	0.929	0.927	0.927		0.863
	SECA5	0.929			0.927	

Table 5 shows the composite reliability (rho-a and rho-c) values and Cronbach's alpha span from 0.841 to 0.927 and 0.839 to 0.927, respectively. Therefore, the composite reliability (rho-A) and Cronbach's alpha values were acceptable for the internal consistency and reliability assessment [17]. The average variance extracted (AVE) values span from 0.568 to 0.863. This finding indicated a good convergent validity for the constructs in the model since the AVE values exceeded the minimum threshold of 0.5 [17].

[66] suggest that all criterion HTMT values must be less than 0.85, while values below 0.9 are acceptable. In addition, the correlations between constructs are known as heterotrait correlations, while the correlation within constructs refers to monotrait correlations [66]. As presented in Table 6, the HTMT values are less than 0.9. Thus, there is discriminant validity (see Table 6).

**Table 6:** Discriminant Validity - HTMT.

	AEXP	BI	CMP	CMPLX	PI	RA	SECA
**AEXP**							
**BI**	0.889						
**CMP**	0.853	0.863					
**CMPLX**	0.437	0.346	0.481				
**PI**	0.737	0.666	0.763	0.362			
**RA**	0.830	0.823	0.880	0.359	0.769		
**SECA**	0.862	0.882	0.842	0.343	0.631	0.780	

These findings suggest that the assessment criteria for validity and reliability of the study's seven (7) constructs presented in the conceptual model are supported.

## 6 Structural model appraisal

The study's findings, presented in Table 7, revealed that the effect sizes (*f*^*2*^) of AEXP (0.068), CMP (0.066), CMPLX (0.020), RA (0.012), and SECA (0.118) on BI are small. In addition, the effect size of PI (0) on BI is nil. Also, the effect size of PI (0.896) on CMP is substantial, while the effect size of PI (0.108) on CMPLX is small. Moreover, the effect size of PI (0.953) on RA is substantial. Finally, the effect size of PI (0.492) on SECA is substantial.

**Table 7:** Hypothesized path's significance effect.

Hypothesized path	Original sample (O)	Sample mean (M)	Standard deviation (STDEV)	T statistics (|O/STDEV|)	(*f*^*2*^)	VIF	(*R*^*2*^)	P values	Significance (p < 0.05)?
PI *→* SECA (H1)	0.574	0.574	0.056	10.313	0.492	1.000	0.330	0	**Yes**
PI *→* RA (H2)	0.699	0.698	0.041	17.231	0.950	1.000	0.488	0	**Yes**
PI *→* CMP (H3)	0.687	0.687	0.041	16.607	0.896	1.000	0.473	0	**Yes**
PI *→* CMPLX (H4)	0.312	0.312	0.07	4.463	0.108	1.000	0.098	0	**Yes**
PI *→* BI (H5)	−0.005	−0.003	0.051	0.102	0.000		0.789	0.918	**No**
SECA *→* BI (H6)	0.336	0.332	0.083	4.05	0.012	3.253		0	**Yes**
RA *→* BI (H7)	0.105	0.114	0.068	1.54	0.012	3.358		0.124	**No**
CMP *→* BI (H8)	0.257	0.259	0.073	3.522	0.066	4.117		0	**Yes**
CMPLX *→* BI (H9)	−0.081	−0.081	0.043	1.868	0.020	1.244		0.062	**No**
AEXP *→* BI (H10a)	0.238	0.228	0.079	2.996	0.068	3.480		0.003	**Yes**
AEXP x SECA *→* BI (H10b)	0.091	0.087	0.061	1.485				0.138	**No**
AEXP x RA *→* BI (H10c)	−0.053	−0.059	0.055	0.958				0.338	**No**
AEXP x CMP *→* BI (H10d)	−0.145	−0.137	0.07	2.092				0.036	**Yes**
AEXP x CMPLX *→* BI (H10e)	0.056	0.055	0.052	1.074				0.283	**No**

**Fig. 2:**
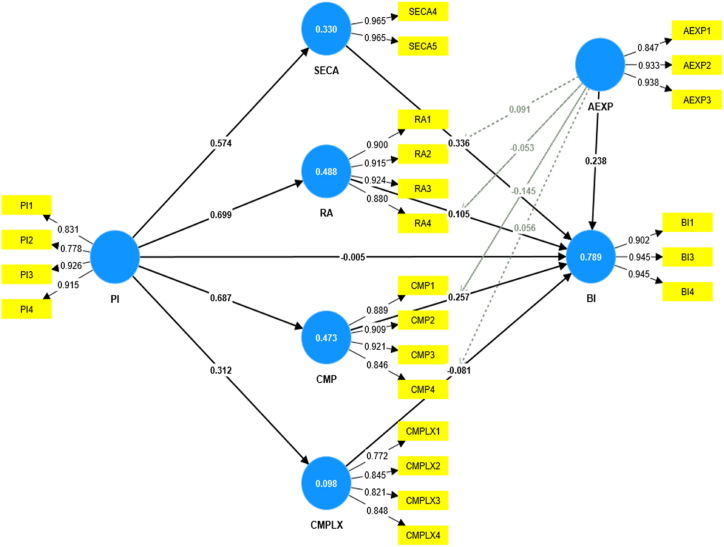
Results of the structural model.

Source: Authors (2024)

Furthermore, as shown in Fig. 2, the path coefficient diagram suggests that the structural model explained 78.9 % of behavioral intention. In addition, the model explained 47.3 % of compatibility, 9 % of complexity, 48.8 % of relative advantage, and 33 % of food security awareness. Therefore, the structural model accounts for 78.9 % predictive accuracy**.**

An importance-performance map analysis (IPMA) (see Table 8) was conducted to determine which constructs are highly important for the target construct but have poor performance [67]. suggest that the IPMA is a trustworthy and valuable analysis in PLS-SEM that extends the normal path coefficients in a more useful manner. Behavioral Intention (BI) was considered the target construct in the IPMA. The latent variable score ranges from 0 to 100, with 0 being the lowest and 100 being the greatest. These scores were rescaled to get the performance scores. The importance scores were obtained from the total direct influence of outcome variables using the structural equation model [17].

**Table 8:** Results of Importance-performance analysis.

Construct	Importance	Performance
**AEXP**	0.238	69.687
**CMP**	0.257	67.630
**CMPLX**	−0.081	52.243
**PI**	0.412	62.429
**RA**	0.105	71.373
**SECA**	0.336	75.862
**Average**	0.211	66.54

**Fig. 3:**
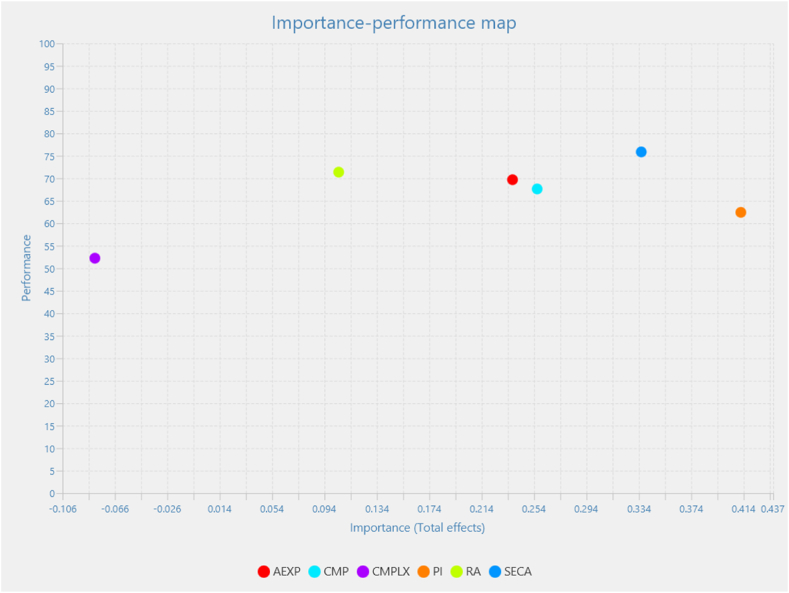
IPMA results.

Source: Authors (2024)

As shown in Fig. 3, as well as informed by the average importance (0.211) and performance (66.54), the following constructs are in the top right quadrant, which is "keep up the good work": agricultural experience, compatibility, and food security awareness. This implies that the study respondents' ratings of the performance of agricultural experience, compatibility, and food security awareness are high and attach higher importance to these factors concerning adopting digital innovation in Ghana's agricultural sector.

Relative advantage is in the top left quadrant, "potential overkill." This implies that the study respondents rated the performance of relative advantage very high but less important in determining the adoption of digital innovation in Ghana's agricultural sector.

Personal innovativeness is in the bottom right quadrant, "focus here." This implies that although the study respondents rated the performance of personal innovativeness low, they attach more importance to it concerning the intention to adopt digital innovation in Ghana's agricultural sector. Finally, complexity is located in the bottom left quadrant, "low priority." This implies that the study respondents rated the performance of complexity as low and less important concerning the intention to adopt digital innovation in Ghana's agricultural sector.

### 6.1 Moderation slope

**Fig. 4:**
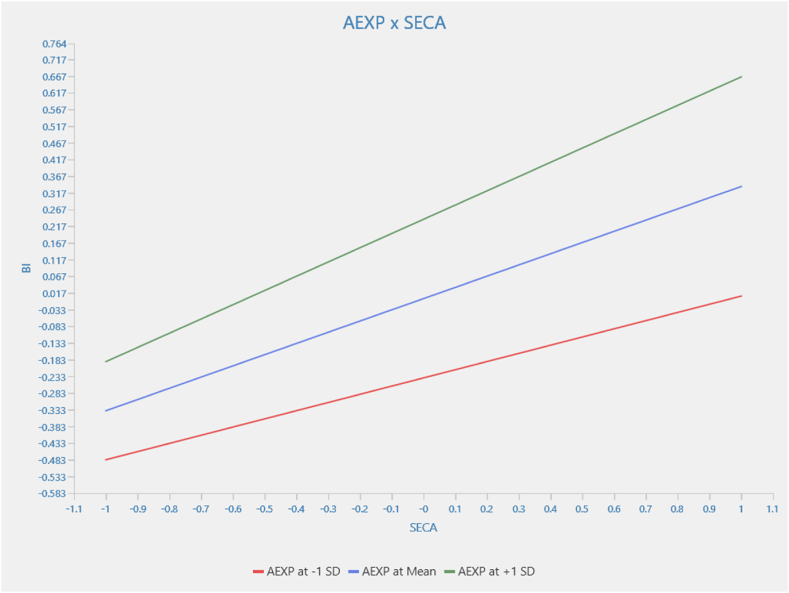
Moderation results for AEXP x SECA → BI.

Source: Authors (2024)

Fig. 4 examines the relationship between food security awareness and behavioral intention, moderated by agricultural experience. However, the relationship between food security awareness and behavioral intention, moderated by agricultural experience, is not statistically significant (β = 0.091; p > 0.05). This finding suggests that the relationship between food security awareness and behavioral intention is consistent regardless of agricultural experience's moderating influence at various levels. This implies that no evidence indicates that agricultural experience affects the strength and direction of the relationship between food security awareness and behavioral intention.

**Fig. 5:**
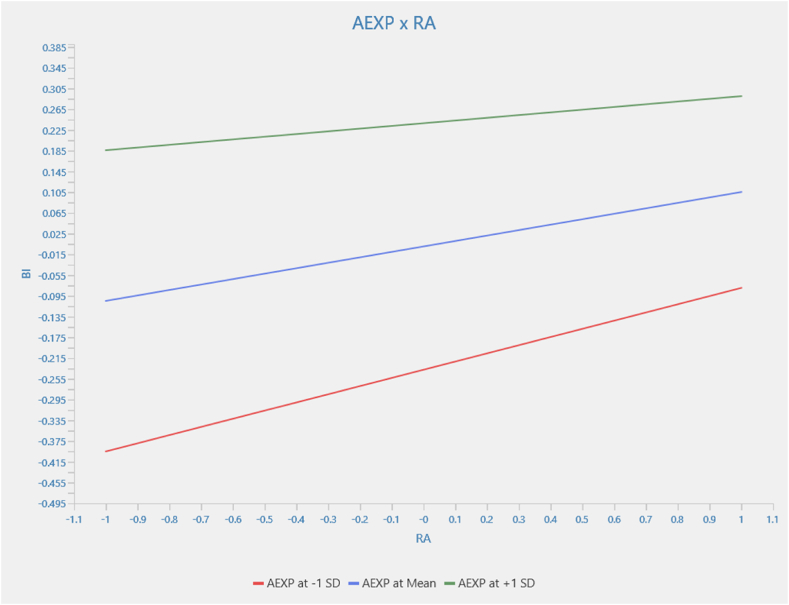
Moderation results for AEXP x RA → BI.

Source: Authors (2024)

Fig. 5 examines the relationship between relative advantage and behavioral intention, moderated by agricultural experience. The interaction term is −0.053. Also, the simple effect of relative advantage on behavioral intention is 0.105. However, the relationship between relative advantage and behavioral intention, moderated by agricultural experience, is not statistically significant (β = −0.053; p > 0.05). This finding suggests that relative advantage consistently affects behavioral intention regardless of the levels of agricultural experience. This implies that no evidence indicates that agricultural experience affects the strength and direction of the relationship between relative advantage and behavioral intention.

**Fig. 6:**
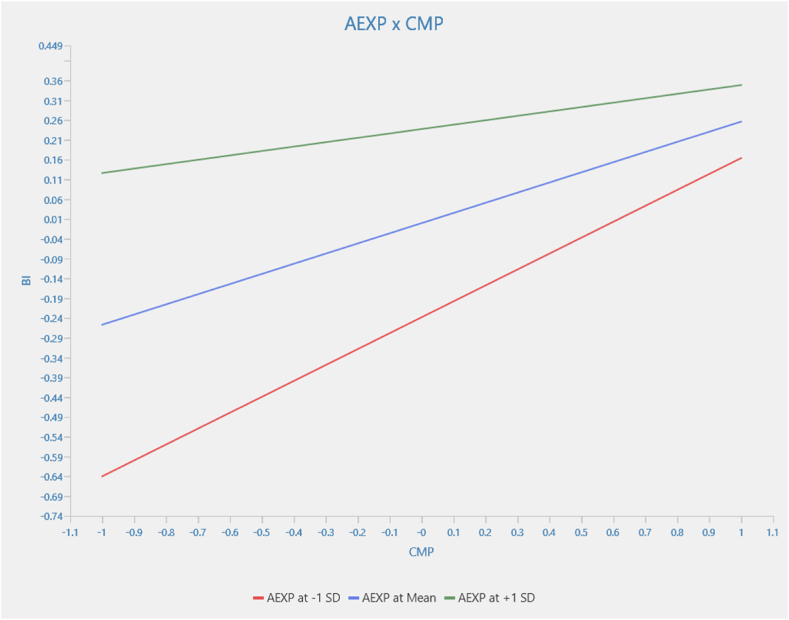
Moderation results for AEXP x CMP → BI.

Source: Authors (2024)

Fig. 6 investigates the relationship between compatibility and behavioral intention, moderated by agricultural experience. The relationship between compatibility and behavioral intention, moderated by agricultural experience, is statistically significant (β = −0.145; p < 0.036). The interaction term negatively affects agricultural experience −0.145. Also, the simple effect of compatibility on behavioral intention is 0.257. This finding implies that for an average level of agricultural experience, the effect of compatibility on behavioral intention is 0.257. For higher levels of agricultural experience, where AEXP increases by one standard deviation, the relationship between compatibility and behavioral intention decreases by the interaction term size. That is, 0.257–0.145 = 0.112. On the other hand, in lower levels of agricultural experience, where AEXP decreases by one standard deviation, the relationship between compatibility and behavioral intention increases. That is, 0.257 + 0.145 = 0.402.

**Fig. 7:**
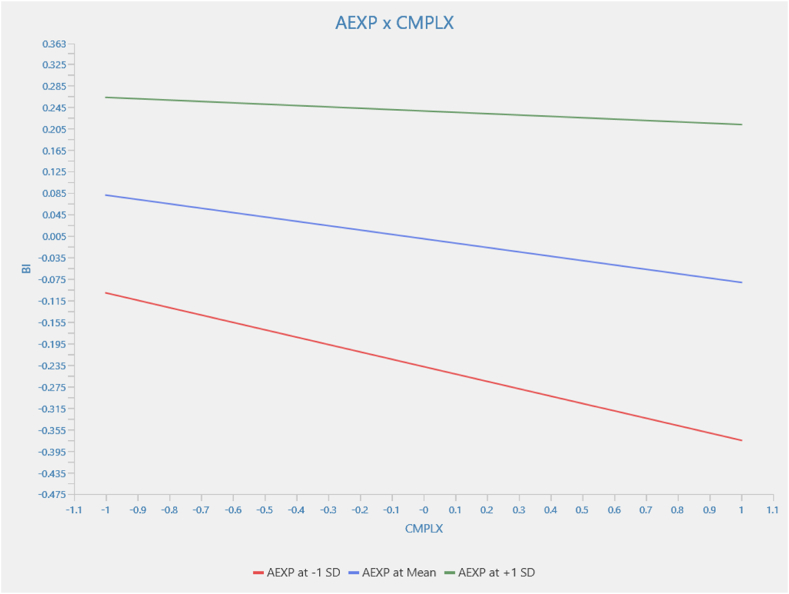
Moderation results for AEXP x CMPLX → BI.

Source: Authors (2024)

Fig. 7 examines the relationship between complexity and behavioral intention, moderated by agricultural experience. The interaction term is 0.056. Also, the simple effect of complexity on behavioral intention is −0.081. However, the relationship between complexity and behavioral intention, moderated by agricultural experience, is not statistically significant (β = −0.056; p > 0.05). This finding suggests that complexity consistently affects behavioral intention regardless of the levels of agricultural experience. This implies that no evidence indicates experience affects the strength and direction of the relationship between complexity and behavioral intention.

## 7 Discussion

Motivated by inadequate understanding and inconclusive findings in the literature on the factors that influence the intention to adopt digital innovation for digital transformation in the agricultural sector in developing countries, this study examined the effect of food security awareness, innovation characteristics, and the moderating role of agricultural experience on behavioral intention to adopt digital innovation in Ghana's agricultural sector. Consequently, this study combined constructs from DOI theory and TAM. The study's findings show that personal innovativeness significantly influences food security awareness (H1). This confirmation implies that individuals with high innovativeness are more likely to adopt digital innovation due to their awareness of its benefits and potential to enhance food security. Prior to this study, the relationship between personal innovativeness and food security awareness had not been investigated. However, existing studies have highlighted the relationship between personal innovativeness and variables such as farm size and agricultural education [4] and value- and-aim system [68]. Thus, this finding can serve as a valuable foundation for further research. Similarly, the study's findings reveal that personal innovativeness has a positive influence on relative advantage (H2), a finding consistent with previous studies [28,36]. This implies that individuals with a higher level of personal innovativeness are more likely to develop positive perceptions about the adoption of digital innovation as having significant advantages over existing agricultural practices. In addition, the study's findings reveal that personal innovativeness has a positive significant influence on compatibility (H3) and complexity (H4). These findings corroborate previous studies [27,28,37]. These results indicate that individuals with higher innovativeness are more likely to perceive digital innovation as aligning with their existing practices and have higher confidence in their ability to use digital innovation, perceiving the complexity of digital innovation as surmountable rather than an obstacle.

Furthermore, the results show that food security awareness has a positive significant effect on behavioral intention (H6). This finding reinforces the assumption that an individual's awareness of food security facilitates the development of more positive intentions due to digital innovation's significant role in promoting food security. The relationship between food security awareness and behavioral intention to adopt digital innovation had not been investigated prior to this study. Thus, this finding can serve as a valuable foundation for further research. Also, the results show that compatibility significantly affects behavioral intention. This finding corroborates [49]. This indicates that individuals who perceive that digital innovation aligns with agricultural practices are more likely to develop positive intentions toward adopting it. Moreover, the finding suggests that agricultural experience has a positive significant effect on behavioral intention (H10a). This result reinforces the assumption that an individual's practical experience in the agricultural sector empowers them to recognize the potential benefits and develop an intention to adopt digital innovation [22]. Also, the study established that agricultural experience moderates the relationship between compatibility and behavioral intention (H10d). This implies that an individual's intention to adopt digital innovation based on how it aligns with agricultural practices and tasks is affected by their level of experience in the agricultural sector. This finding can serve as a valuable premise for further studies since this relationship had not been explored prior to this study.

On the contrary, the study findings revealed that personal innovativeness does not significantly affect behavioral intention (H5), a finding inconsistent with previous studies [35,37,38]. It can be inferred that individuals' innovativeness does not inform their intention to adopt digital innovation. Also, the study's findings suggest that relative advantage does not significantly affect the behavioral intention to adopt digital innovation (H7), a finding inconsistent with previous studies [43,44]. Thus, individuals in Ghana's agricultural sector do not necessarily develop intentions to adopt digital innovation to obtain a relative advantage. Moreover, the finding suggests that complexity does not significantly affect behavioral intention (H9). It can be inferred that perceived complexity does not matter in developing intentions to adopt digital innovation. This finding is inconsistent with [49,51], and [50].

Furthermore, the findings reveal that agricultural experience does not moderate the following relationships: food security awareness and behavioral intention (H10b), relative advantage and behavioral intention (H10c), and complexity and behavioral intention (H10e). These relationships have not been investigated prior to this study. Given that digital innovation is considered a means to ensure food security, individuals are more likely to develop positive intentions to adopt it, irrespective of their agricultural experience. Moreover, the intention to adopt digital innovation is affected by its ability to address the needs of individuals, irrespective of their agricultural experience. Similarly, the nature and functionalities of digital innovations may appear complex or less complex to individuals in Ghana's agricultural sector, irrespective of their agricultural experience.

### 7.1 Theoretical and practical implications

By investigating the factors that influence digital innovation in Ghana's agricultural sector, the research offers the following theoretical contributions. To begin, there is a paucity of research that examines the adoption of digital innovation in Ghana's agricultural sector using a dyadic model. Informed by DOI and TAM, this research extends the applicability of these theories to investigate the adoption of digital innovation adoption in Ghana's agricultural sector. Though DOI and TAM have been combined to investigate the adoption of digital innovation in various contexts (e.g., Refs. [69,70]), it has not been applied in the context of Ghana's agricultural sector. Considering that the conceptual model of the study demonstrates 78.9 % predictive accuracy, this research advances knowledge since TAM usually accounts for approximately 53 % variance [71].

In addition, this research provides a dyadic perspective to understanding the factors that influence the adoption of digital innovation in Ghana's agricultural sector by combining DOI and TAM. Given that there is a significant difference between digital innovation characteristics and intention to adopt, integrating these two theories facilitated the comprehensive investigation of digital innovation characteristics, contextual factors, and intention to adopt. Moreover, despite the wide use of DOI and TAM in digital innovation adoption research (e.g., Ref. [72]), such studies have not adequately accounted for personal innovativeness, food security awareness, and agricultural experience as fundamental predictors of the adoption of digital innovation. Integrating DOI and TAM suggests that the adoption of digital innovation in Ghana's agricultural sector depends on the characteristics of digital innovation, behavioral intention, agricultural experience, and food security awareness. Furthermore, this study provides an alternative perspective by demonstrating that personal innovativeness, relative advantage, and complexity do not significantly affect behavioral intention to adopt digital innovation. In addition, agricultural experience does not moderate the relationship between food security awareness, relative advantage, and complexity on behavioral intention. Hence, this research provides an alternative perspective that serves as a premise for further investigation. These alternative perspectives provided by this research are deemed essential contributions to knowledge advancement.

### 7.2 Practical implications

This research also offers practical implications. From the findings of the importance-performance analysis, this research reveals that personal innovation is the most important factor in determining the adoption of digital innovation in Ghana's agricultural sector. This is followed by food security awareness, compatibility, agricultural experience, and relative advantage. This indicates that regulators and policymakers in Ghana's agricultural sector should concentrate on developing a culture that facilitates innovativeness among the individuals involved in the agricultural sector. Also, policymakers and regulators can focus on training and resource allocation to improve innovation skill development, innovative idea generation, and the development of a conducive environment that supports and rewards innovative solutions in the agricultural sector. For instance, the District Department of Agriculture can implement agri-hubs to facilitate innovation idea development and collaboration between the government, academic institutions and agri-tech companies. In addition, leveraging digital innovation development via mobile-based agricultural services can improve innovation skill acquisition and resource allocation due to the high penetration of mobile penetration in Ghana.

Furthermore, policymakers and regulators should emphasize the need to adopt digital innovations that address specific challenges and needs faced by Ghana's agricultural sector. Thus, regulators can collaborate with local farmers, agricultural extension officers, financial institutions, and agricultural innovation developers to co-create context-specific solutions that can efficiently and effectively improve resilience, productivity, and food security.

### 7.3 Policy implications

The findings of the study suggest that personal innovativeness and food security awareness can be enhanced through policy review. This indicates that regulators and policymakers should emphasize implementing policies that support personal innovativeness and food security awareness in Ghana's agricultural sector. For instance, regulators and policymakers can develop digital innovation support programs, and food security awareness campaigns to enlighten and sensitize stakeholders on the strategic role of digital innovation in ensuring food security. In addition, policy development should focus on funding, training, and equipping stakeholders and farmers in the agricultural sector to become more innovative to facilitate the adoption of digital innovation.

In addition, regulators should facilitate the development of partnerships, collaboration, and digital innovation transfer programs between agri-tech companies, government agencies, research institutions, and farmers to drive digital innovation adoption. For example, regulators can develop partnerships with telecommunication companies to leverage their nationwide mobile network infrastructure to facilitate mobile-agricultural service diffusion to remote areas.

Furthermore, policymakers should establish a regulatory framework that encourages the adoption of digital innovation in Ghana's agricultural sector. Such regulatory frameworks should not impede the adoption of innovative practices among stakeholders in Ghana's agricultural sector.

### 7.4 Study limitations

Though this research offers essential insights into digital innovation adoption for digital transformation in Ghana's agricultural sector, which sheds light on the interaction between digital innovation adoption factors and an enabling environment to engender digital transformation, there are some research limitations. First, this research focused on the agricultural sector in Ghana, a low- and middle-income country. Hence, there is the possibility that the findings of this research may not apply to high-income or developed countries' contexts due to different socioeconomic and political factors that undergird these contexts. In addition, Ghana's agricultural sector is characterized by other actors such as agricultural extension officers, financial institutions, farmers, agri-tech companies, merchants, etc. However, this research focused on the perspectives of regulators of Ghana's agricultural sector, specifically the Department of Agriculture, under the auspices of MoFA. The narrow scope limits the perspective to governmental perspectives. This scope could be increased to account for broader sectoral insights such as market dynamics, economic conditions, digital agriculture leadership, digital trust, and digital divide, which could significantly influence digital innovation adoption in Ghana's agricultural sector. Future studies could investigate the effect of these variables to enhance our understanding of the factors that influence digital innovation adoption from a broader sectoral perspective.

## 8 Conclusion

This study sought to examine the factors that influence users' intention to adopt digital innovation in Ghana's agricultural sector by proposing a dyadic model that was informed by combining DOI theory and TAM. The model demonstrates good explanatory power, which attests to its robustness in predicting the intention to adopt digital innovations in Ghana's agricultural sector. Also, this is arguably the first study in information systems research to investigate the effect of food security awareness and the moderating role of agricultural experience on behavioral intention. The study's findings confirm that personal innovativeness positively affects food security awareness, relative advantage, compatibility, and complexity. Moreover, the findings proffered new insights that food security awareness, compatibility, and agricultural experience positively affect behavioral intention. Furthermore, findings revealed an insignificant effect of personal innovativeness, relative advantage, and complexity on behavioral intention. Furthermore, the study's findings confirm the moderating role of agricultural experience on the relationship between compatibility and behavioral intention. These findings advanced knowledge of personality characteristics, innovation characteristics, and contextual factors that influence digital innovation adoption in Ghana's agricultural sector. Furthermore, the variables of food security awareness and agricultural experience examined in this study are novel in the digital innovation literature. This study reported on the effects of these variables on behavioral intention.

## CRediT authorship contribution statement

**David Aboagye-Darko:** Writing – review & editing, Writing – original draft, Methodology, Investigation, Formal analysis, Conceptualization. **Peter Mkhize:** Writing – review & editing, Supervision.

## Data availability statement

Data will be made available on request. To request data, please write to the corresponding author.

## Declaration of competing interest

The authors declare that they have no known competing financial interests or personal relationships that could have appeared to influence the work reported in this paper.
