# Supplementary material for: Unearthing the determinants of digital innovation adoption in the agricultural sector: The role of food security awareness and agricultural experience
Source: Heliyon. 2025 Jan 3;11(1):e41695. doi: 10.1016/j.heliyon.2025.e41695 (PMC11760315; doi:10.1016/j.heliyon.2025.e41695)
Supplement: Multimedia component 1 [file mmc1.docx]

**QUESTIONNAIRE**

**SECTION A**

**Demographic information**

**Kindly indicate “x” in the appropriate box**

1. **Gender:** Male [ ] Female [ ]
2. **Age:**

18 – 25 [ ], 26 – 30 [ ], 31 – 35 [ ], 36 – 40 [ ], 41 – 45 [ ], 46 – 50 [ ],

51 – 55 [ ], 56 – 65 [ ]

1. **Qualification:**

Bachelor’s Degree [ ]

Masters [ ]

PhD [ ]

Professional program [ ]

Other: ………………….

1. Which digital innovation do you use? Please, select as many as possible

Mobile applications [ ]

Mobile payment platforms [ ]

Cloud computing [ ]

Social media platform [ ]

Artificial intelligence platforms [ ]

Virtual assistants [ ]

Internet of Things platforms [ ]

Blockchain platforms [ ]

Others: ………

**SECTION B**

**Kindly indicate the most appropriate box. Choose from a range of choices from Strongly Disagree to Strongly Agree.**

(Strongly Disagree = 1, Disagree = 2, Somewhat Disagree = 3, Neutral = 4, Somewhat agree = 5, Agree = 6, Strongly Agree = 7)

**PERSONAL INNOVATIVENESS (Adapted from Agarwal and Prasad, 1998)**

I think of new ways of applying digital innovation to agriculture if I learn about it

I am among the first to try out digital innovations for agricultural practices

I enjoy examining digital innovations introduced to the agricultural sector

I am usually eager to try out digital innovations for agricultural practices

**RELATIVE ADVANTAGE (Adapted from Rogers, 2003)**

Adopting digital innovation gives me more advantages than traditional methods in agriculture

Adopting digital innovation is more convenient for my agricultural practices than traditional methods

Adopting digital innovation enhances my agricultural processes more efficiently than traditional methods

Adopting digital innovation is more effective for managing my agricultural tasks compared to traditional methods

**COMPATIBILITY (Adapted from Rogers, 2003)**

Integrating digital innovation into my agricultural practices aligns with all aspects of my work

Digital innovation aligns well with my preferred way of managing agricultural activities

Adopting digital innovation harmonizes with my existing agricultural work style

Integrating digital innovation into my work activities aligns with optimizing task outcomes

**COMPLEXITY (Adapted from Rogers, 2003)**

Adopting digital innovation for my agricultural practices appears difficult to understand

Adopting digital innovation designed for agriculture challenges my comprehension

Understanding how to use digital innovation in agriculture demands significant effort

Adopting digital innovation for my agricultural work activities requires substantial time

**BEHAVIOURAL INTENTION (Adapted from Davis, 1989)**

I intend to increase my usage of digital innovation for agricultural practices in the future

I am most likely to incorporate digital innovation into my agricultural practices

I will always try to use digital innovation for my agricultural work activities

I intend to continue using digital innovation for my agricultural activities

**AGRICULTURAL EXPERIENCE (Adapted from Anum et al., 2022)**

I have extensive work experience in the agricultural sector

I have an in-depth understanding of agricultural practices and techniques

I have adequate experience in managing and promoting agricultural development

I have participated in various agricultural workshops and training programs

**FOOD SECURITY AWARENESS (Adapted from Mabe et al., 2021)**

I am aware of the food security issues affecting my community

I stay informed about food security challenges and trends

Ensuring food security is important to me in my decision-making

I have an in-depth understanding of what food security entails

I actively participate in and support food security initiatives
